# Fully Automatic Localization and Segmentation of 3D Vertebral Bodies from CT/MR Images via a Learning-Based Method

**DOI:** 10.1371/journal.pone.0143327

**Published:** 2015-11-23

**Authors:** Chengwen Chu, Daniel L. Belavý, Gabriele Armbrecht, Martin Bansmann, Dieter Felsenberg, Guoyan Zheng

**Affiliations:** 1 Institution for Surgical Technology and Biomechanics, University of Bern, 3014 Bern, Switzerland; 2 Charité - University Medicine Berlin, Centre of Muscle and Bone Research, Campus Benjamin Franklin, Free University & Humboldt-University Berlin, 12200 Berlin, Germany; 3 Centre for Physical Activity and Nutrition Research, School of Exercise and Nutrition Sciences, Deakin University Burwood Campus, Burwood VIC 3125, Australia; 4 Institut für Diagnostische und Interventionelle Radiologie, Krankenhaus Porz Am Rhein gGmbH, 51149 Köln, Germany; Henry Jackson Foundation, UNITED STATES

## Abstract

In this paper, we address the problems of fully automatic localization and segmentation of 3D vertebral bodies from CT/MR images. We propose a learning-based, unified random forest regression and classification framework to tackle these two problems. More specifically, in the first stage, the localization of 3D vertebral bodies is solved with random forest regression where we aggregate the votes from a set of randomly sampled image patches to get a probability map of the center of a target vertebral body in a given image. The resultant probability map is then further regularized by Hidden Markov Model (HMM) to eliminate potential ambiguity caused by the neighboring vertebral bodies. The output from the first stage allows us to define a region of interest (ROI) for the segmentation step, where we use random forest classification to estimate the likelihood of a voxel in the ROI being foreground or background. The estimated likelihood is combined with the prior probability, which is learned from a set of training data, to get the posterior probability of the voxel. The segmentation of the target vertebral body is then done by a binary thresholding of the estimated probability. We evaluated the present approach on two openly available datasets: 1) 3D T2-weighted spine MR images from 23 patients and 2) 3D spine CT images from 10 patients. Taking manual segmentation as the ground truth (each MR image contains at least 7 vertebral bodies from T11 to L5 and each CT image contains 5 vertebral bodies from L1 to L5), we evaluated the present approach with leave-one-out experiments. Specifically, for the T2-weighted MR images, we achieved for localization a mean error of 1.6 mm, and for segmentation a mean Dice metric of 88.7% and a mean surface distance of 1.5 mm, respectively. For the CT images we achieved for localization a mean error of 1.9 mm, and for segmentation a mean Dice metric of 91.0% and a mean surface distance of 0.9 mm, respectively.

## 1 Introduction

In clinical routine, lower back pain (LBP) caused by spinal disorders is reported as a common reason for clinical visits [1, 2]. Both computed tomography (CT) and magnetic resonance (MR) imaging technologies are used in computer assisted spinal diagnosis and therapy support systems. MR imaging becomes the preferred modality for diagnosing various spinal disorders such as spondylolisthesis and spinal stenosis [3]. At the same time, CT images are required in specific applications such as measuring bone mineral density of vertebral bodies (VBs) for diagnosing osteoporosis [4, 5]. For all these clinical applications, localization and segmentation of VBs from CT/MR images are prerequisite conditions.

In this paper, we address the two challenging problems of localization and segmentation of VBs from a 3D CT image or a 3D T2-weighted Turbo Spin Echo (TSE) spine MR image. The localization aims to identify the location of each VB center, where segmentation handles the problem of producing binary labeling of VB/non-VB regions for a given 3D image. For vertebra localization, there exist both semi-automatic methods [3, 6, 7] and fully automatic methods [8–13]. For vertebra segmentation, both 2D MR image-based methods [6, 13, 14] and 3D CT/MR image-based methods [3, 4, 7, 15–21] are introduced in literature. These methods can be roughly classified as model based methods [3, 15–18] and graph theory (GT) based methods [4, 6, 7, 13, 14, 19, 20].

**Localization of VBs**: A simple and fast way to achieve the vertebra localization is done by introducing user interactions and user inputs [3, 6, 7]. In literature, approaches which use either one user-supplied seed point [3, 6] or multiple user-defined landmarks [7] are introduced for VB localization.

In contrast to the semi-automatic methods, there also exist automatic localization methods using single/multi-class classifier [9, 10] or model-based deformation [11, 12]. Schmidt et al. [9] proposed a method to localize spine column from MR images using a multi-class classifier in combination with a probabilistic graphical model. Another similar work using multi-class classifier and graphical model was reported by Oktay et al. [10]. This work was further extended by Lootus et al. [11] to detect VB regions in all 2D slices of a 3D MR volume. Zhan et al. [12] proposed a model-based method, where a robust hierarchical algorithm was presented to detect arbitrary numbers of vertebrae and inter-vertebral discs (IVDs).

Recent advancement of machine learning-based methods provides us another course of efficient localization methods [8, 13]. Both Huang et al. [13] and Zukić et al. [15] proposed to use AdaBoost-based methods to detect vertebral candidates from MR images. To identify and label VBs from 3D CT data, Glocker et al. [8] proposed a supervised, random forest (RF) regression-based method [22, 23]. Another regression based framework was introduced in [24], where a data-driven regression algorithm was proposed to tackle the problem of localizing IVD centers from 3D T2 weighted MRI data. Both Glocker et al. [8] and Chen et al. [24] further integrated prior knowledge of inter-relationship between neighboring objects using Hidden Markov Model (HMM), for the purpose of elimination of potential ambiguity caused by the repetitive nature between neighboring vertebrae.

**Segmentation of VBS**: For vertebra segmentation, model-based methods [3, 15–18] were introduced in literature. Zukić et al. [3, 15] presented a semi-automatic method, which used the segmented vertebral model to deduce geometric features for diagnosis of the spinal disorders. The authors reported an average segmentation accuracy of 78%. Klinder et al. [16, 17] proposed to use non-rigid deformation to guide statistical shape model (SSM) fitting and reported an average segmentation error of 1.0 mm when evaluated on CT images. In [18], volumetric shapes of VB was deterministically modeled using super-quadrics by introducing 31 geometrical parameters. The segmentation was then performed by optimizing the parameters to match the 3D parametrized model with a given image. When validated on MR images, this method obtained an average segmentation error of 1.85mm.

GT based methods [4, 6, 7, 13, 14, 19, 20] are now widely used in vertebra segmentation. Among the methods in this category, there exist methods in the form of normalized cut [13, 14]. For example, Carballido-Gamio et al. [14] applied the normalized cut to segment T1-weighted MR images. Huang et al. [13] improved this method by proposing an iterative algorithm. Although potentially this method could be applied to 3D MR images, Huang et al. [13] only evaluated their method on 2D sagittal MR slices.

There also exist GT based methods in the form of graph cut [4, 5, 7]. Aslan et al. [4, 5] presented a graph cut method to segment lumbar and thoracic vertebrae. Another related method was presented by Ayed et al. [7], which incorporated feature-based constraints into graph cut optimization framework [25, 26]. Evaluated on 15 2D mid-sagittal MR slices, this method achieved an average 2D Dice overlap coefficient of 85%.

Recent literature witnessed the successful application of another type of GT based methods which were usually formulated as graph theory-based optimal surface search problems [6, 19, 20]. Yao et al. [19] proposed to achieve the vertebra segmentation with a spinal column detection and partition procedure. More recently, following the idea introduced in [27, 28], both *square-cut* and *cubic-cut* methods were proposed. The square-cut method works only on 2D sagittal slice of MRI while the cubic-cut method can be used for 3D spinal MR image segmentation. Despite the above mentioned differences, these two methods essentially use a similar process to first construct a directed graph for a target structure and then to search for the boundary of the target structure from the constructed graph by applying a graph theory-based optimal boundary search process [27, 28].

In this paper, inspired by the work presented in [8, 23, 29, 30], we propose a learning-based, unified random forest regression and classification framework to tackle the problems of fully automatic localization and segmentation of VBs from a 3D CT image or a 3D T2-weighted TSE MR image. More specifically, in the first step, the localization of a 3D VB in a given image is solved with random forest regression where we aggregate votes from a set of randomly sampled 3D image patches to get a probability map. The resultant probability map is then further regularized by HMM to eliminate potential ambiguity caused by the neighboring VBs. The output from the first step allows us to define a region of interest (ROI) for the segmentation step, where we use random forest classification to estimate the likelihood of a voxel in the ROI being foreground or background. The estimated likelihood is then combined with the prior probability, which is learned from a set of training data, to get the posterior probability of the voxel. The segmentation of the target VB is then done by a binary thresholding of the estimated probability.

## 2 Method

**Fig 1:**
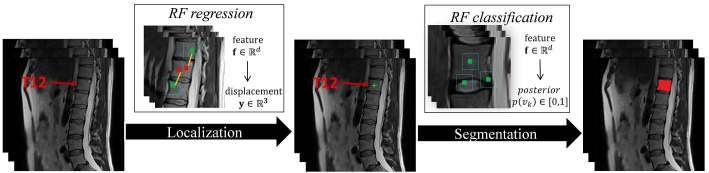
The flowchart of the proposed VB localization and segmentation method.

The study was approved by the Ethical Committee of the Charité University Medical School Berlin. Fig 1 gives an overview of the proposed method. In the following sections, details are given for each stage of the present method.

### 2.1 Localization of vertebral bodies

**Fig 2:**
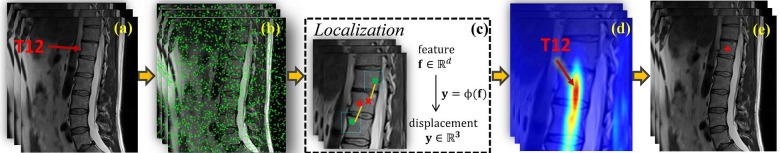
An example for detecting the center of VB T12 via RF regressions on a 3D MR image. (a) A target image. (b) Centers of randomly sampled patches on the target image. (c) Each patch gives a single vote to predict the center of VB T12. (d) Response volume calculated using Gaussian transform. (f) Selected mode from the response volume as the predicted center.

For each VB, we separately train and apply a RF regressor [23] to estimate its center. Fig 2 gives an example for detecting the center of VB T12 from a 3D MR image.

#### 2.1.1 Training

During the training, we have a set of annotated training images where in each training image, the boundaries of multiple VB regions are manually delineated beforehand. The geometrical center of each delineated VB region is then calculated as the associated ground-truth. From each training image and for one VB, we sample a set of 3D training image patches around the ground-truth VB center. Each sampled patch is represented by its visual feature **f**_*i*_ and its displacement **d**_*i*_. Let us denote all the sampled patches from all training images as {*v*_*i*_ = (**f**_*i*_,**d**_*i*_)}, where *i* = 1…*N*. The goal is then to learn a mapping function $\phi :R^{d_{f}}\to R^{3}$ from the feature space to the displacement space. In principle, any regression method can be used. In this paper, similar to [23], we utilize the random forest regressor.

#### 2.1.2 Detection

Once the regressor is trained, given an unseen image, we randomly sample another set of 3D image patches $\left\{v_{j}^{\prime }=\left(f_{j}^{\prime },c_{j}^{\prime }\right)\right\}$ (Fig 2b), where *j* = 1…*N*′ all over the unseen image (or an region of interest if an initial guess of the VB center is known). Similarly, **f** ^**′**^_*j*_ is the visual feature and **c** ^**′**^_*j*_ is the center of the *j*th sampled patch, respectively (Fig 2c). Through the learned mapping *φ*, we can calculate the predicted displacement **d** ^**′**^_*j*_ = *φ*(**f** ^**′**^_*j*_), and then **y**_*j*_ = **d** ^**′**^_*j*_+**c** ^**′**^_*j*_ becomes the prediction of the center position by a single patch $v_{j}^{\prime }$. Note that each tree in the random forest will return a prediction. Therefore, supposing that there are *T* trees in the forest, we will get *N*′ × *T* predictions. These individual predictions are very noisy, but when combined, they approach an accurate prediction. By aggregating all these predictions we will get a soft probability map called *response volume* (Fig 2) which gives, for every voxel of the unseen image, its probability of being the VB center. The probability aggregation using Gaussian transform is time-consuming when executed on a 3D image data. Thus, we adapt an improved fast Gaussian transform (IFGT) [31] based probability aggregation algorithm introduced in our previous work [32], aiming to accelerate the detection algorithm. For completeness, below we briefly summarize the details of our fast probability aggregation algorithm.

#### 2.1.3 Fast probability aggregating

We consider each single vote as a small Gaussian distribution $N∼(\bar{d_{j}^{\prime }},\Sigma \left(d_{j}^{\prime }\right))$, where $\bar{d_{j}^{\prime }}$ and $\Sigma \left(d_{j}^{\prime }\right)=diag\left(\sigma_{j,x}^{2},\sigma_{j,y}^{2},\sigma_{j,z}^{2}\right)$ are mean and covariance. For detection of each VB center, *N*′ × *T* predictions are produced and aggregated using multivariate Gaussian transform as follows.

$$\begin{array}{c} G\left(y_{i}\right)=\sum_{j}^{N^{\prime }\times T}\frac{1}{\sqrt{{\left(2\pi \right)}^{3}\left|\Sigma \left(d_{j}^{\prime }\right)\right|}}exp{(-\frac{1}{2}\left(d_{y_{i}}-\bar{d_{j}^{\prime }}\right)}^{T}\left({\Sigma \left(d_{j}^{\prime }\right))}^{-1}\left(d_{y_{i}}-\bar{d_{j}^{\prime }}\right)\right) \end{array}$$ (1)

where $d_{y_{i}}=y_{i}-c_{j}^{\prime }$, $y_{i}$ is a voxel in target image and $c_{j}^{\prime }$ is the center of patch *j*. For detecting each VB center, such a calculation will finally require prohibitively expensive computation time of *O*(*M* × *N*′ × *T*) on a 3D image with *M* voxels. In our previous work [32], we propose to approximate Eq (1) by:

$$\begin{array}{c} G\left(y_{i}\right)=\sum_{j}^{N^{\prime }\times T}W_{j}\cdot e^{\left(∥d_{y_{i}}-\bar{d_{j}^{\prime }}{∥}^{2}/h^{2}\right)} \end{array}$$ (2)

Here we rewrite the Eq (1) by introducing a constant kernel width of *h*, and we move the variances out of the exponential part by introducing a weight *W*_*j*_. With such an approximation, we develop a fast strategy using IFGT algorithm [31] to calculate the response images with highly reduced computational time of *O*(*M*+*N*′ × *T*) for detecting each VB center.

#### 2.1.4 Fast Visual Feature Computing

**Fig 3:**
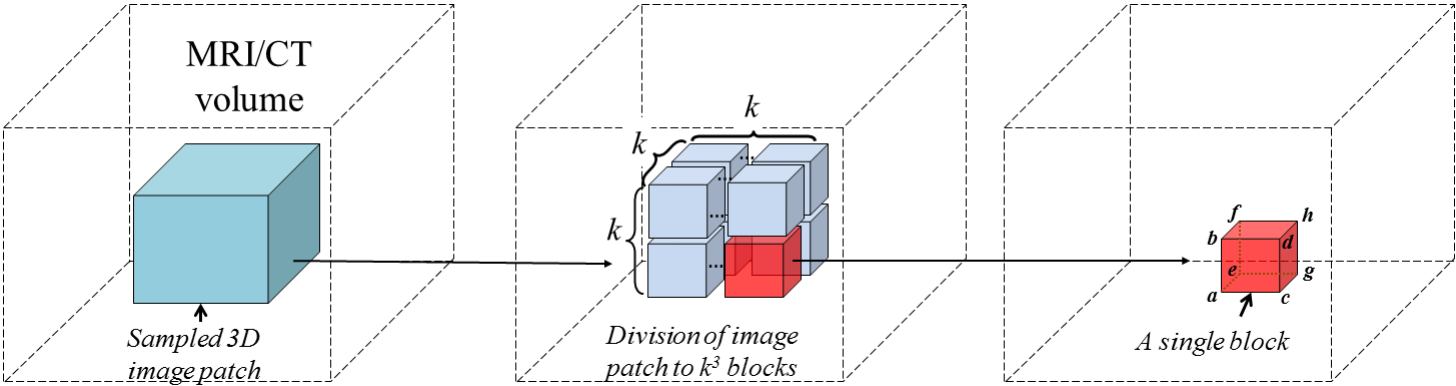
A schematic illustration on how to compute the visual feature of a sampled 3D image patch for RF training and regression. **Left**: a sub-volume is sampled from a MRI/CT volume. **Middle**: we divide the sampled image patch into *k* × *k* × *k* blocks. **Right**: for each block, we compute its mean and variance using the integral image technique.

The neighborhood intensity vector of 3D CT/MR image patches are used for computing the visual feature. Specifically, we evenly divided a sampled patch from a CT or a MR image into *k* × *k* × *k* blocks (Fig 3), and the mean intensities in each block are concatenated to form a *k* ^3^ dimensional feature and normalized to unit *L*_1_ norm. We further compute the variance of intensities in each block if those patches are sampled from a CT image, aiming to deal with the diffused intensity values in CT images for achieving accurate localization. Thus for image patches extracted from a MR image, we compute a *k* ^3^ dimensional feature and for image patches extracted from a CT image we compute a 2*k* ^3^ dimensional feature.

In designing the visual feature for MR images, we are aware of the work on inter-scan MR image intensity scale standardization [33] as well as intra-scan intensity inhomogeneity correction or bias correction [34, 35] and their applications in the scoliotic spine [36]. However, considering the relatively small imaging field of view in our study and the fact that the bias field is said to be smooth and slowly varying and is composed of low frequency components only [36], we choose to normalize our feature to accommodate for both intra-scan and inter-scan intensity variations: our feature vector is the concatenation of mean image intensities in different blocks within a local neighborhood (3D image volume), and then we divide the vector by its *L*_1_ norm to make it sum up to one. This makes the feature insensitive to global or low frequency local intensity shifting, because the feature vector is not dependent on the absolute intensity in the neighborhood and what matters is the relative difference of intensities in different blocks. This makes our feature sensitive to gradient rather than to the absolute intensity values, which may also explain why we can extend such a visual feature from MR images to CT images.

To accelerate the feature extraction within each block, we use the well-known integral image technique as introduced in [37]. Details about how to compute the integral image of a quantity can be found in [37]. The quantity can be the voxel intensity value or any arithmetic computation on the intensity value. Advantage of using integral image lies in the fact that once we obtain an integral image of the quantity over the complete MRI/CT volume, the sum of the quantity in any sub-volume can be calculated quickly in constant time O(1) no matter how big the size of the volume is [37]. Here we assume that we already computed the integral images of the voxel intensity *I* and the integral images of the squared voxel intensity *S* of the complete MRI/CT volume using the technique introduced in [37]. We then compute the mean *E*[*X*] and the variance *Var*(*X*) of the intensity value of any block (Fig 3, right) as:

$$\begin{array}{c} \left\{ \begin{array}{c} E[X]=\left(I(h)-I(d)-I(f)-I(g)+I(b)+I(c)+I(e)-I(a)\right)/N_{v};\\ E[X^{2}]=\left(S(h)-S(d)-S(f)-S(g)+S(b)+S(c)+S(e)-S(a)\right)/N_{v};\\ Var(X)=E[X^{2}]-{\left(E[X]\right)}^{2}. \end{array}.\right. \end{array}$$ (3)

where $\left\{a,\ldots h\right\}\in R^{3}$ are the eight vertices of the block and *N*_*v*_ is the number of voxels within the block.

#### 2.1.5 Coarse-to-fine strategy

**Fig 4:**
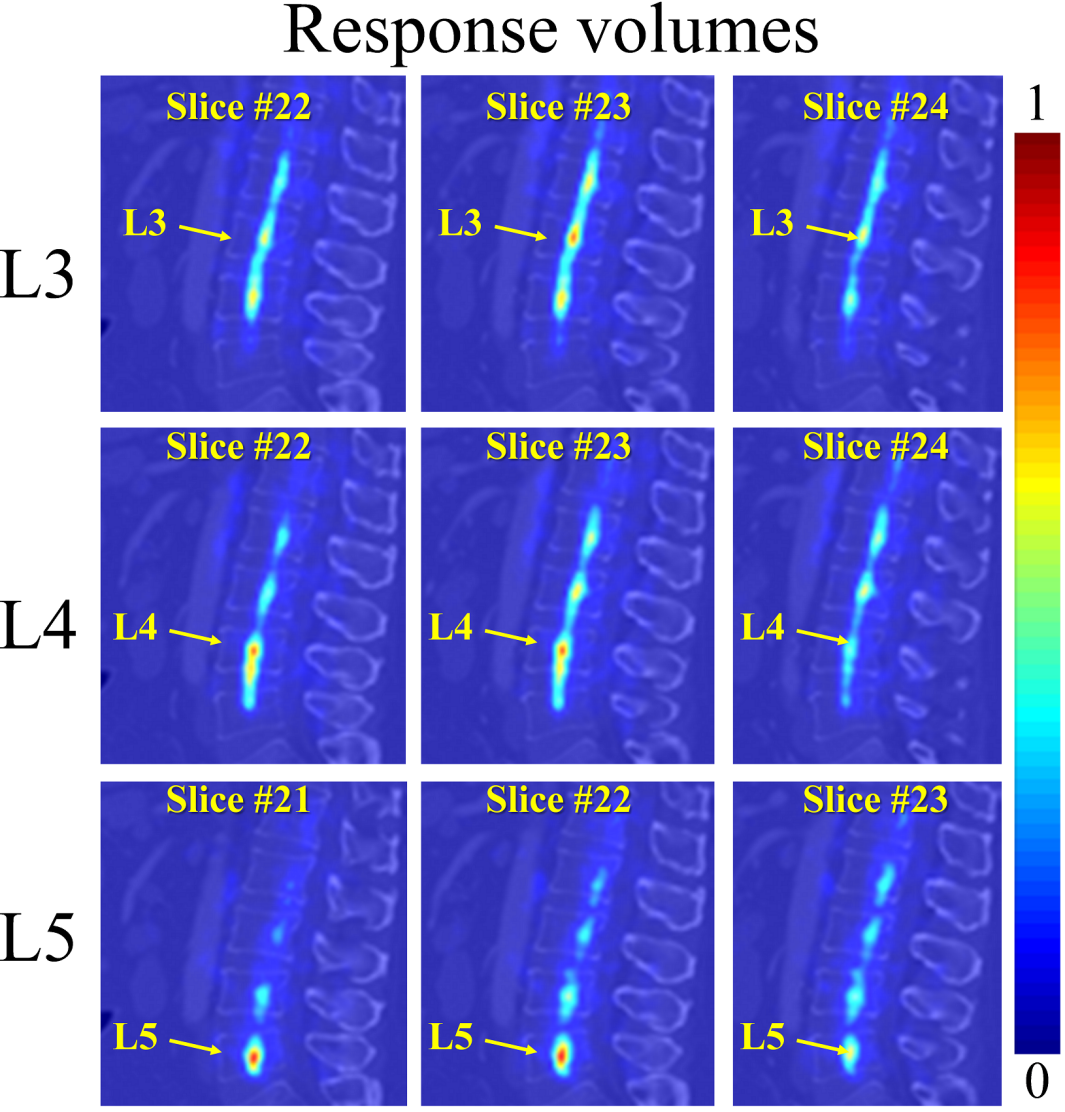
Initial estimation of the VB centers on one test CT image. The estimation is done in a coarse resolution. The response volume of L3, L4, and L5 are visualized in each row, with 3 randomly selected 2D sagittal slices. The diffused probability distribution is observed in the response volumes due to the repetitive pattern of the VBs.

**Fig 5:**
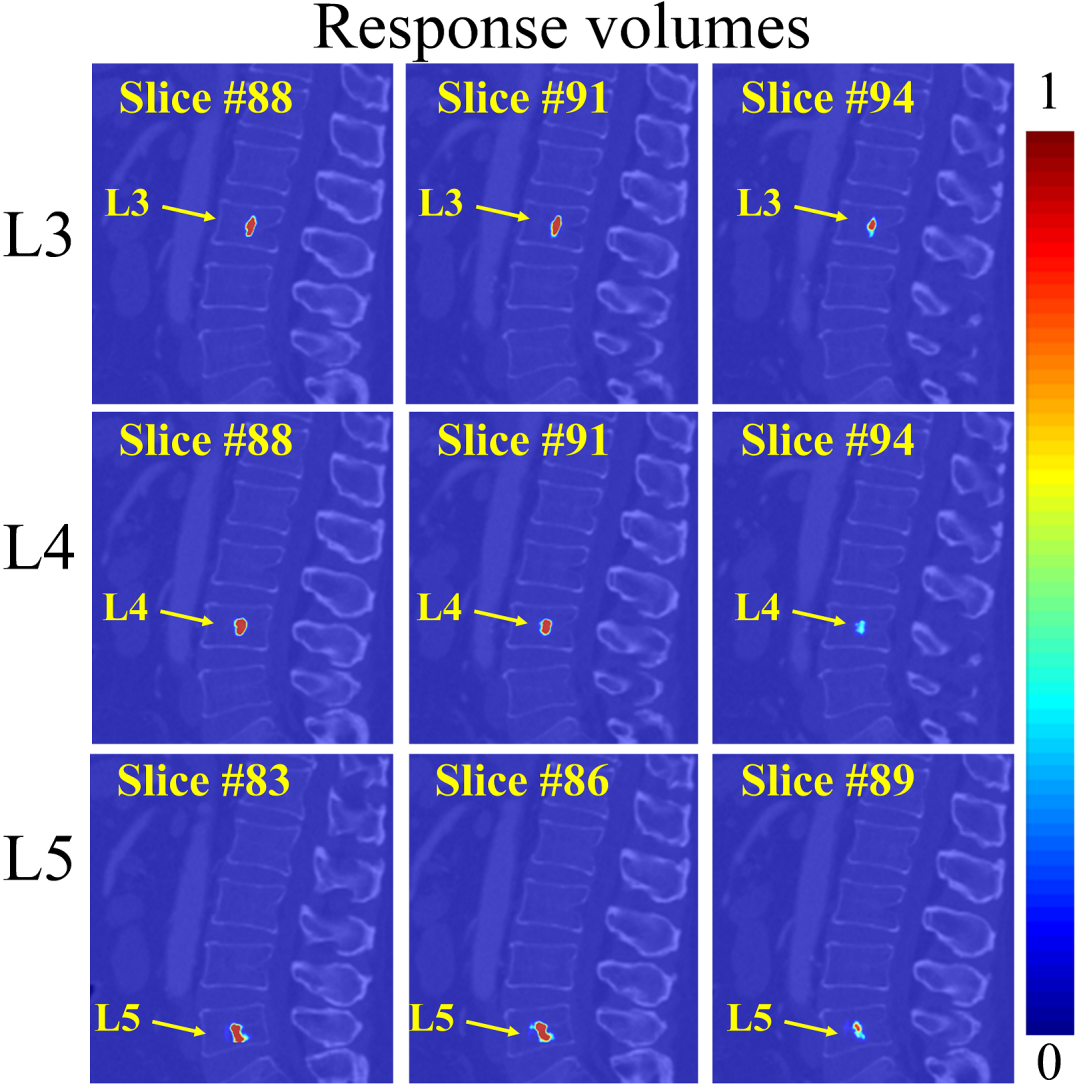
The fine-tuning step for localization of the estimated VB centers on the same test CT image used in Fig 4. The response volume of L3, L4, and L5 are visualized in each row, with 3 randomly selected 2D sagittal slices. The fine-tuning is performed only in a reduced local region around the initial guess obtained from the first step. Thus, the associated probabilities in the response volume are concentrated to a small region.

We conduct VB center detection with a two-step coarse-to-fine strategy executed in two different resolutions. In the coarse step, using sampled patches all over a down-sampled image (During the down-sampling, we maintain the inter-slice resolution but down-sample the intra-slice resolution with a scale factor of 1/4 along each direction. Please note, our MR image slices are parallel to the YZ plane of the data coordinate system while our CT image slices are parallel to the XY plane of the data coordinate system.), an initial guess of each VB center position is estimated. This initial detection may have potential ambiguities due to the repetitive pattern of VBs and the large detection region (see Fig 4 for examples). This is further improved by an additional HMM model based optimization to encode the prior geometric constraints between neighboring centers as in [8]. In the fine-tuning step, we try to localize a VB center in the original image resolution but only in a reduced small local region around the initial guess obtained from the first step (Fig 5). Below we present the detailed algorithm on HMM based regularization of the VB center detection.

Assuming that we are interested in *m* VBs, after we separately apply trained *m* RF regressors to the target image, we can compute *m* response volumes *I*_*i*_(*v*)_*i* = 1…*m*_, one for each VB region. We regularize the result by using spine shape prior, where the shape prior is learned from a given set of training images by considering the inter-VB relations. Since the spine is in a cord structure with sequential VBs, we exploit the relative position of adjacent VBs for generating spine shape prior that captures the conditional probabilities over the VB center positions. For the *i*th and the (*i*+1)th VBs, we collect the relative offset of their centers from the training images, and approximate the offsets by a Gaussian distribution *G*_*i*, *i*+1_(.|*μ*_*i*,*i*+1_,*Σ*_*i*,*i*+1_) with the mean *μ*_*i*,*i*+1_ and variance *Σ*_*i*,*i*+1_. Then, the transitional probability of two VB centers on the test image is given by:

$$\begin{array}{c} p_{i,i+1}\left(c_{i+1}|c_{i}\right)=G_{i,i+1}\left(c_{i+1}-c_{i}|\mu_{i,i+1},\Sigma_{i,i+1}\right) \end{array}$$ (4)

where *c*_*i*_ and *c*_*i*+1_ are the center positions of the *i*th and the (*i*+1)th VBs, respectively.

On the other hand, the observation probability is simply given by the associated response volume:

$$\begin{array}{c} p_{i}\left(c_{i}\right)=I_{i}\left(c_{i}\right) \end{array}$$ (5)

The optimal sequence of VB centers are thus given by maximizing the following joint probability:

$$\begin{array}{c} {\text{argmax}}_{c_{1}\cdots c_{m}}p_{1}\left(c_{1}\right)p_{1,2}\left(c_{2}|c_{1}\right)\cdots p_{m-1,m}\left(c_{m}|c_{m-1}\right)p_{m}\left(c_{m}\right) \end{array}$$ (6)

This can be solved by dynamic programming on the image grids.

### 2.2 Segmentation of vertebral bodies

**Fig 6:**
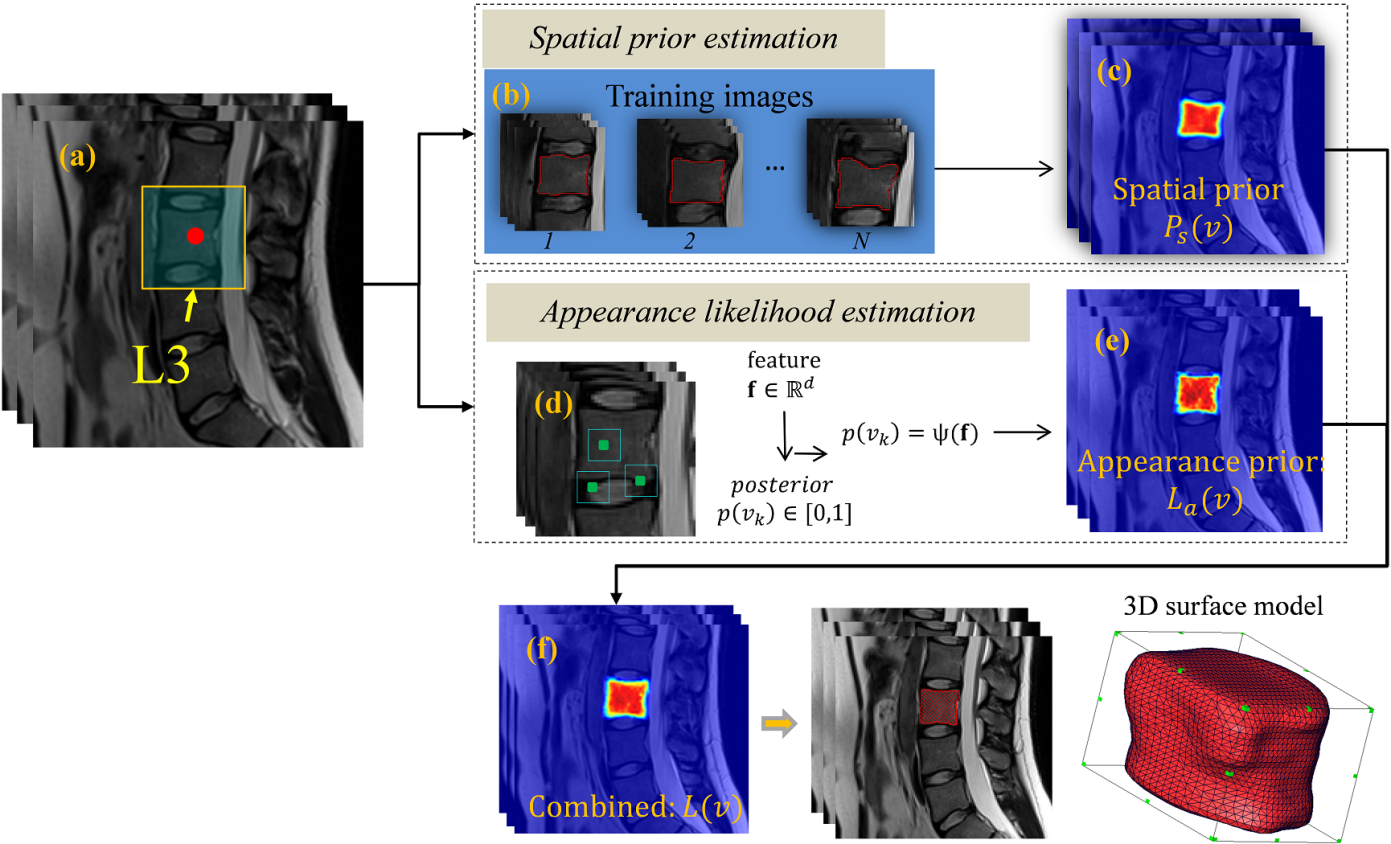
Segmentation of vertebral body in its ROI. (a): ROI of VB L3. (b)-(e): Segmentation procedure using spatial prior ((b) and (c)) and RF soft classification based appearance likelihood ((d) and (e)) to estimate the posterior probability (f). The final segmentation results are obtained by a thresholding on the estimated posterior probability.

The segmentation of VBs is separately done in the defined ROI around each detected VB center as shown in Fig 6. For each voxel *v* in the defined ROI, we first compute an appearance likelihood *L*_*a*_(*v*) (Fig 6, d and e) and a spatial prior *P*_*s*_(*v*) (Fig 6b and 6c), where *L*_*a*_(*v*) is estimated using the RF soft classification algorithm described below and *P*_*s*_(*v*) is estimated via a Parzen window method. In our method, for every voxel in the ROI of a detected VB, we first compute its spatial prior *P*_*s*_(*v*). The resultant prior *P*_*s*_(*v*) serves as a good pre-filter of the potential foreground voxels, where only for those voxels with *P*_*s*_(*v*)>0.1, we compute its appearance likelihood *L*_*a*_(*v*). Once *P*_*s*_(*v*) and *L*_*a*_(*v*) are calculated for every voxel, we get the combined posterior probability map *L*(*v*) (Fig 6d) as:

$$\begin{array}{c} L\left(v\right)=\alpha \cdot P_{s}\left(v\right)+\beta \cdot L_{a}\left(v\right) \end{array}$$ (7)

With the posterior probability map *L*(*v*), for each voxel in the ROI of the VB, its probability of being the foreground is given. The final binary segmentation is derived by thresholding the probability map with *L*(*v*)≥0.5 and only keeping the largest connected component. Below we give the details of using RF soft classification method to estimate the appearance likelihood *L*_*a*_(*v*).

#### 2.2.1 RF classification based appearance likelihood estimation: Training

Similar to the localization step, given a set of manually labeled training images, we randomly sample a set of 3D training patches {*v*_*k*_ = (**f**_*k*_, *l*_*k*_)}_*k* = 1…*M*_, where **f**_*k*_ is the visual feature and *l*_*k*_ = {1,0} is the foreground/background label of the center of a sampled patch, being in the ROI of specified VB. The sampled training patches can be divided into positive training patches if *l*_*k*_ = 1 and negative training patches if *l*_*k*_ = 0. Using both the sampled positive and negative training patches, our task is then to learn a mapping function $\psi :R^{d_{f}}\to p\left(v_{k}\right)\in \left[0,1\right]$ from the feature space to the probability space. We utilize classification forest to train the mapping function. For each forest, we suppose there are *T*_*s*_ trees. Please note we use the same visual feature as we used in the localization step (Sec. 2.1.4).

#### 2.2.2 RF classification based appearance likelihood estimation: prediction

Once the mapping function *ψ* is learned, for each voxel *v* in the ROI of a detected VB region, we first calculate its visual feature **f**_*v*_. Through the learned mapping *ψ*, for every voxel in the ROI, we estimate its appearance likelihood of being the foreground/background. Note that each tree in the classification forest will return a prediction *p*_*t*_(*l*_*v*_|**f**_*v*_)∈[0, 1], where *l*_*v*_ = {0,1}. Combining all these *T*_*s*_ predictions allows us to compute a reliable posterior likelihood for each voxel *v* as follows.

$$\begin{array}{c} L_{a}\left(v\right)=p\left(l_{v}|f_{v}\right)=\frac{1}{T_{s}}\sum_{t}^{T_{s}}p_{t}\left(l_{v}|f_{v}\right) \end{array}$$ (8)

### 2.3 Implementation details

A Matlab implementation (It is freely available from “http://ijoint.istb.unibe.ch/VB/index.html”) of the present method is tested using the experiment setup that will be described in Sec. 3.1. All parameters used in our experiments are summarized in Table 1. The visual features for sampled 3D CT/MR image patches are calculated following the description in Sec. 2.1.4. Specifically, we evenly divide a sampled patch into 4 × 4 × 4 blocks. Thus for each image patch extracted from a MR image, we compute a 64 dimensional feature and for each image patch extracted from a CT image we compute a 128 dimensional feature.

**Table 1:** Parameters used in our experiments.

Modality	Parameters and values	Comments
MRI	*d*_*f*_ = 64	Dimension of computed features for each sampled image patch
	Patch size: 16 × 20 × 20 Voxels	Patch size used for localization of VB centers at the coarse step
	Patch size: 8 × 40 × 40 Voxels	Patch size used for localization of VB centers at the fine-tuning step
CT	*d*_*f*_ = 128	Dimension of computed features for each sampled image patch
	Patch size: 20 × 20 × 12 Voxels	Patch size used for localization of VB centers at the coarse step
	Patch size: 80 × 80 × 32 Voxels	Patch size used for localization of VB centers at the fine-tuning step
For both modalities	Patch size: 8 × 8 × 8 Voxels	Patch size used for VB segmentation
	*N* = 15000	Number of sampled training image patches for localization, at both coarse and fine-tuning steps
	*N*′ = 10000	Number of test image patches, both in coarse and fine-tuning steps
	*M* = 12000	Number of training image patches for segmentation
	*W*_*j*_ = 1	See Eq (2)
	*h* = 1.5	See Eq (2)
	*α* = 0.4	See Eq (7)
	*β* = 0.6	See Eq (7)

In the localization stage, for both the coarse detection step and the fine-tuning step we sample *N* = 15000 training image patches from training images while for test we sample a set of *N*′ = 10000 image patches from the test image. In the segmentation step, for each VB region we sample *M* = 12000 training image patches. We use different sizes for image patches extracted from MRI and CT data: 1) For a MR image, where its slices are parallel to the YZ plane of the data coordinate system, the patch sizes in different stages are chosen as follows. In the localization stage, a patch size of 16 × 20 × 20 voxels is used for the coarse detection of the VB centers and for the fine-tuning step we use a patch size of 8 × 40 × 40 voxels. For the segmentation stage, we use a patch size of 8 × 8 × 8 voxels. 2) For a CT image, where its slices are parallel to the XY plane of the data coordinate system, we choose the patch sizes in different stages as follows. In the localization stage, a patch size of 20 × 20 × 12 voxels is used in the coarse detection step and for the fine-tuning step we use a patch size of 80 × 80 × 32 voxels. For the segmentation stage, we use a patch size of 8 × 8 × 8 voxels.

For both CT and MR images, we empirically chose *W*_*j*_ = 1 and *h* = 1.5 in Eq (2), *α* = 0.4 and *β* = 0.6 in Eq (7).

## 3 Experiments and Results

### 3.1 Experimental design

We validate our method on two openly available CT/MRI datasets: 1) The first dataset contains 23 3D T2-weighted turbo spin echo MR images from 23 patients and the associated ground truth segmentation. They are freely available from “http://dx.doi.org/10.5281/zenodo.22304”. Each patient was scanned with 1.5 Tesla MRI scanner of Siemens (Siemens Healthcare, Erlangen, Germany) with following protocol to generate T2-weighted sagittal images: repetition time is 5240 ms and echo time is 101 ms. All the images are sampled to have the same sizes of 39 × 305 × 305 voxels. The voxel spacings of all the images are sampled to be 2 × 1.25 × 1.25 mm^3^. In each image 7 VBs T11-L5 have been manually identified and segmented, resulting in 161 labeled VBs in total. 2) The second dataset contains 10 3D spine CT images and the associated ground truth segmentation [21]. They are freely available from “http://spineweb.digitalimaginggroup.ca/spineweb/index.php?n=Main.Datasets”. The sizes of these CT images are varying from 512 × 512 × 200 to 1024 × 1024 × 323 voxels with intra-slice resolutions between 0.28245 mm and 0.79082 mm and inter-slice distances between 0.725 mm and 1.5284 mm. We further resample all the images into the same voxel spacing of 0.5 × 0.5 × 1.0 mm^3^, which simplifies the implementation. For each CT image, 5 VBs L1-L5 have been manually annotated, resulting in 50 VBs in total.

Using the two openly available MRI and CT datasets, we evaluated our VB localization and segmentation method with the following 4 experiments:

1. **VB localization on MRI dataset**. In this experiment, we evaluated the present VB localization method on the 23 T2-weighted MR images.
2. **VB localization on CT dataset**. In this experiment, we evaluated the present VB localization method on the 10 CT images.
3. **VB Segmentation on MRI dataset**. In this experiment, we evaluated the present VB segmentation method on the 23 T2-weighted MR images.
4. **VB Segmentation on CT dataset**. In this experiment, we evaluated the present VB segmentation method on the 10 CT images.

In each one of the above mentioned 4 experiments, a leave-one-out study was conducted where each time one patient data was chosen for test and the remaining data were used for training.

### 3.2 Evaluation metrics

We propose to use five different metrics to evaluate the performance of the present method, two for localization stage and three for segmentation stage.

For evaluation of the localization performance, we use the following two metrics:

1. **Mean localization distance (MLD) with standard deviation (SD)**
  We first compute the localization distance *R* for each VB center using
  $$\begin{array}{c} R=\sqrt{{\left(\Delta x\right)}^{2}+{\left(\Delta y\right)}^{2}+{\left(\Delta z\right)}^{2}} \end{array}$$ (9)
  where *Δx* is the absolute difference between *X* axis of the identified VB center and the VB center calculated from the ground truth segmentation, *Δy* is the absolute difference between *Y* axis of the identified VB center and the ground truth VB center, and *Δz* is the absolute difference between *Z* axis of the identified VB center and the ground truth VB center.
  The equations of MLD and SD are then defined as follows:
  $$\begin{array}{c} \text{MLD}=\frac{\sum_{i=1}^{N_{I}}\sum_{j=1}^{M_{VB}}R_{ij}}{N_{c}}\;\;\text{and}\;\;\text{SD}=\sqrt{\frac{\sum_{i=1}^{N_{I}}\sum_{j=1}^{M_{VB}}{\left(R_{ij}-\text{MLD}\right)}^{2}}{N_{c}}} \end{array}$$ (10)
  where *N*_*c*_ is the total number of VBs, *N*_*I*_ is the number of patient data, and *M*_*VB*_ is the number of target VBs in each image.
2. **Successful detection rate with various ranges of accuracy**
  If the absolute difference between the localized VB center and the ground truth center is no greater than *t* mm, the localization of this VB is considered as a successful detection; otherwise, it is considered as a false localization. The equation of the successful localization rate *P*_*t*_ with accuracy of less than *t* mm is formulated as follows
  $$\begin{array}{c} P_{t}=\frac{\text{number}\;\text{of}\;\text{accurate}\;\text{VB}\;\text{localization}}{\text{number}\;\text{of}\;\text{VBs}} \end{array}$$ (11)

For evaluating the segmentation performance, we use the following three metrics:

1. **Dice overlap coefficients (Dice)**
  This metric measures the percentage of correctly segmented voxels. Dice [38] is computed by
  $$\begin{array}{c} \text{Dice}=\frac{2|A\cap B|}{\left|A|+|B\right|}\times 100\% \end{array}$$ (12)
  where *A* is the sets of foreground voxels in the ground-truth data and *B* is the corresponding sets of foreground voxels in the segmentation result, respectively. Larger Dice metric means better segmentation accuracy.
2. **Average Absolute Distance (AAD)** This metric measures the average absolute distance from the ground truth VB surface and the segmented surface. To compute the AAD, we first generate surface meshes from segmented binary VB volumes. For each vertex on the surface model derived from the automatic segmentation, we found its closest distance to the surface model derived from the associated ground-truth segmentation. The AAD was then computed as the average of distances of all vertexes. Smaller average absolute distance means better segmentation accuracy.
3. **Hausdorff Distance (HSD)** This metric measures the Hausdorff distance [39] between the ground truth VB surface and the segmented surface. To compute the HSD, we use the same surface models generated for computing the AAD. Smaller Hausdorff distance means better segmentation accuracy.

### 3.3 Experimental Results

#### 3.3.1 Localization results on MRI data

Table 2 presents MLD with SD when the present method was evaluated on 23 T2-weighted MR images. The localization error (average of the 7 VBs) of each test image as well as overall MLD, SD and median value of all 23 MR images are shown in this table. A localization accuracy of 1.6 ± 0.9 mm was found, which were regarded to be accurate enough for the purpose of defining ROI for each VB region.

**Table 2:** Average localization error (MLD with SD: mm) when evaluated on 23 3D MR images.

Average localization error of vertebral body centers (mm)													
#1	#2	#3	#4	#5	#6	#7	#8	#9	#10	#11	#12	Mean ± STD	Median
2.6	1.9	2.5	2.9	1.6	1.1	1.6	1.3	1.7	0.9	1.5	1.1		
#13	#14	#15	#16	#17	#18	#19	#20	#21	#22	#23		1.6 ± 0.9	1.6
2.2	1.6	2.1	1.1	1.5	1.4	1.7	1.4	1.7	1.3	1.2			

Table 3 gives the results of successful detection rates of the present method with different accuracy range *t* = 2.0 mm, 4.0 mm, and 6.0 mm, respectively. Given the specified accuracy range *t* = 2.0 mm, our method successfully detected 76.4% VBs. The successful detection rate is changed to 97.5% when we set *t* to 4.0 mm and all the 161 VBs are successfully detected when we set accuracy range *t* to 6.0 mm.

**Table 3:** Successful detection rate with various ranges of accuracy when evaluated on 23 3D MR images. In the first row, number of successfully detected VBs are given, and in the second row the successful detection rate are shown.

	t = 2.0mm	t = 4.0mm	t = 6.0mm
Number of successfully detected VBs	123	157	161
Successful detection rate (%)	76.4	97.5	100.0

#### 3.3.2 Localization results on CT data

Table 4 presents MLD with SD when the present method was evaluated on 10 CT images. The localization error (average of the 5 VBs) of each test image as well as overall MLD, SD and median value are presented in this table. An overall localization accuracy of 1.9 ± 1.5 mm was found, which were regarded to be accurate enough for the purpose of defining ROI for each VB region.

**Table 4:** Average localization error (MLD with SD: mm) when evaluated on 10 3D CT data.

Average localization error of vertebral body centers (mm)											
#1	#2	#3	#4	#5	#6	#7	#8	#9	#10	Mean ± STD	Median
1.6	1.9	0.6	1.4	2.5	2.1	2.9	1.4	1.9	2.7	1.9 ± 1.5	1.8

Table 5 gives the results of successful detection rates of the present method with different accuracy range *t* = 2.0 mm, 4.0 mm, and 6.0 mm, respectively. Given the specified accuracy range *t* = 2.0 mm, our method successfully detected 58% VBs out of 50 VBs. The successful detection rate is changed to 94% when *t* is set to 4.0 mm and this rate is further changed to 96% when we set *t* to 6.0 mm.

**Table 5:** Successful detection rate with various ranges of accuracy when evaluated on 10 3D CT images. In the first row, number of successfully detected VBs are given, and in the second row the successful detection rate are shown.

	t = 2.0mm	t = 4.0mm	t = 6.0mm
Number of successfully detected VBs	29	47	48
Successful detection rate (%)	58.0	94.0	96.0

#### 3.3.3. Segmentation results on MRI data

For quantitative evaluation of the present method on the 23 MR images, the Dice, AAD, and HSD between automatic segmentation and the ground-truth segmentation are calculated over both 3D volumes and 2D mid-sagittal slices. The reason why we also calculate results on 2D mid-sagittal slice is because some of the existing methods are only evaluated on 2D MR images (e.g., [6, 13]). Table 6 presents the Dice, AAD, and HSD (average of the 7 VBs) of each test image as well as overall mean, std and median values of Dice, AAD, and HSD when calculated on 2D mid-sagittal slice. Table 7 presents the Dice, AAD, and HSD of each image as well as overall mean, std and median values of Dice, AAD, and HSD when calculated on 3D volumes. In summary, we achieved a mean Dice of 92.0±3.4%, a mean AAD of 1.0±0.4 mm, and a mean HSD of 4.5±1.4 mm when calculated on 2D mid-sagittal slices. For 3D evaluation, we achieved a mean Dice of 88.7±2.9%, a mean AAD of 1.5±0.2 mm, and a mean HSD of 6.4±1.2 mm.

**Table 6:** Segmentation results when the present method was evaluated on 23 3D MR images with a leave-one-out experiment. The results are calculated on 2D mid-sagittal slices.

Dice overlap coefficient on 2D mid-sagittal slice (%)													
#1	#2	#3	#4	#5	#6	#7	#8	#9	#10	#11	#12	Mean ± STD	Median
89.6	87.7	89.2	86.6	93.6	93.1	91.0	92.9	93.0	92.5	93.6	93.9		
#13	#14	#15	#16	#17	#18	#19	#20	#21	#22	#23		92.0 ± 3.4	92.6
93.0	90.9	90.4	93.1	94.9	93.5	92.1	94.4	90.8	92.7	93.2			
Average absolute distance on 2D mid-sagittal slice (mm)													
#1	#2	#3	#4	#5	#6	#7	#8	#9	#10	#11	#12	Mean ± STD	Median
1.5	1.4	1.4	1.8	0.8	0.9	1.1	0.8	0.9	0.9	0.8	0.8		
#13	#14	#15	#16	#17	#18	#19	#20	#21	#22	#23		1.0 ± 0.4	0.9
0.7	1.2	1.3	0.9	0.6	0.8	0.9	0.7	1.1	1.1	0.8			
Hausdorff distance on 2D mid-sagittal slice (mm)													
#1	#2	#3	#4	#5	#6	#7	#8	#9	#10	#11	#12	Mean ± STD	Median
5.9	5.1	6.3	7.6	2.7	3.4	5.6	3.7	3.5	3.5	3.7	4.9		
#13	#14	#15	#16	#17	#18	#19	#20	#21	#22	#23		4.5 ± 1.4	4.9
5.5	3.7	6.6	4.2	3.0	3.3	5.1	1.8	5.0	5.5	5.0			

**Table 7:** Segmentation results when the present method was evaluated on 23 3D MR images with a leave-one-out experiment. The results are calculated on 3D volumes.

Dice overlap coefficient on 3D volume (%)													
#1	#2	#3	#4	#5	#6	#7	#8	#9	#10	#11	#12	Mean ± STD	Median
88.6	86.9	86.7	85.1	88.4	89.2	88.2	87.1	88.3	89.5	89.5	89.9		
#13	#14	#15	#16	#17	#18	#19	#20	#21	#22	#23		88.7 ± 2.9	89.3
86.6	88.5	88.0	87.3	91.8	91.0	87.3	91.2	87.9	91.9	91.7			
Average absolute distance on 3D volume (mm)													
#1	#2	#3	#4	#5	#6	#7	#8	#9	#10	#11	#12	Mean ± STD	Median
1.7	1.6	1.8	1.9	1.5	1.5	1.6	1.6	1.6	1.4	1.4	1.4		
#13	#14	#15	#16	#17	#18	#19	#20	#21	#22	#23		1.5 ± 0.2	1.5
1.5	1.6	1.7	1.6	1.3	1.3	1.5	1.3	1.5	1.3	1.3			
Hausdorff distance on 3D volume (mm)													
#1	#2	#3	#4	#5	#6	#7	#8	#9	#10	#11	#12	Mean ± STD	Median
6.5	8.3	8.1	8.3	6.7	6.2	7.3	6.2	5.9	5.1	7.8	5.6		
#13	#14	#15	#16	#17	#18	#19	#20	#21	#22	#23		6.4 ± 1.2	6.2
6.1	5.5	7.0	6.9	6.1	4.6	7.4	5.0	6.6	4.3	4.7			

**Fig 7:**
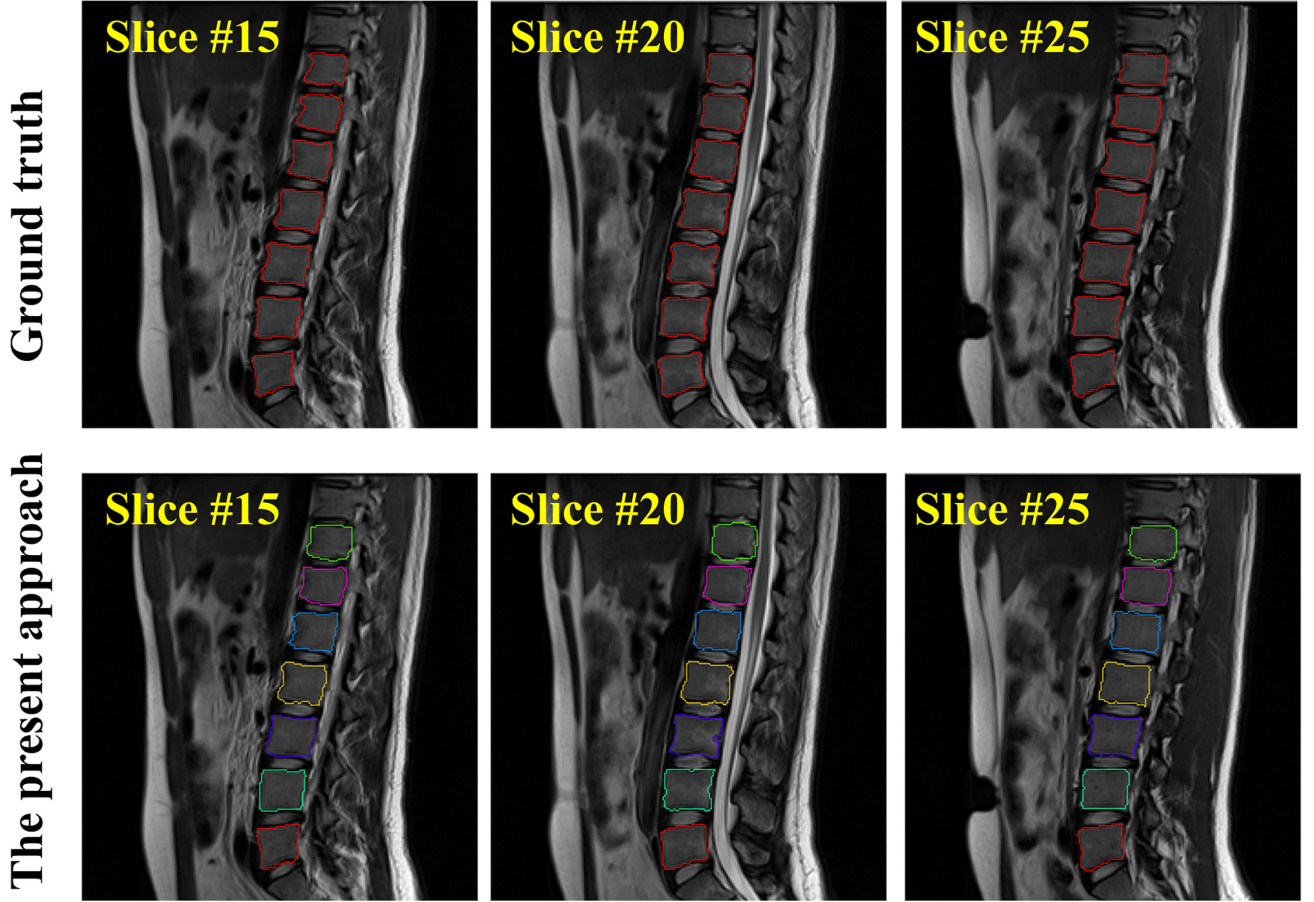
Segmentation results on one test MR image visualized in 2D sagittal slices. The automatic segmentation (the bottom row) are compared with the ground-truth segmentation (the top row).

**Fig 8:**
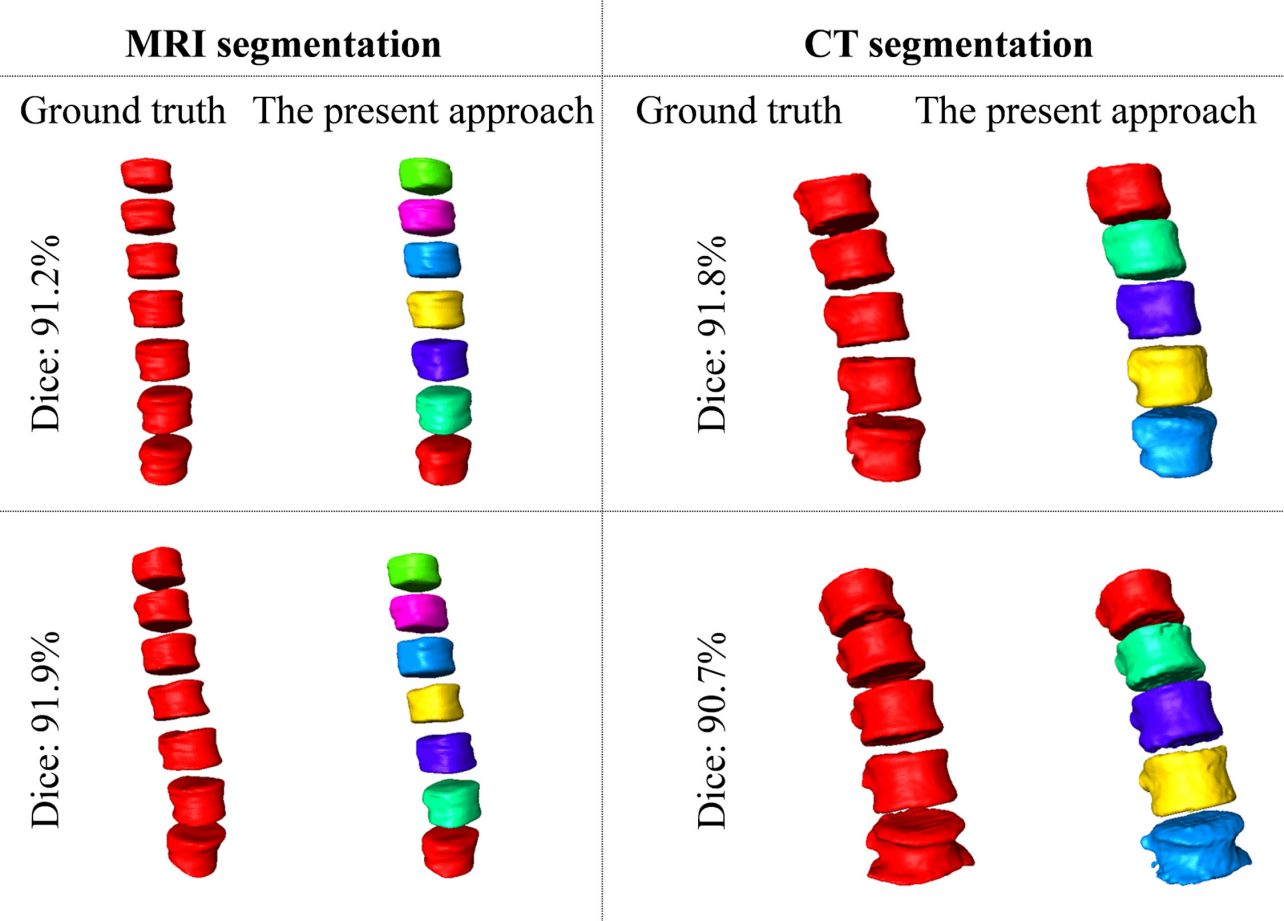
Segmentation results visualized with 3D surface models. Images on the left side show the segmentation results on 2 3D MR test images and images on the right side present the segmentation results on 2 3D CT images. It is worth to note that the second CT data (bottom right image) shows osteophytes in some of the VBs but our method successfully identified and segmented all the 5 VB regions in this CT data with a Dice of 90.7%.

In Fig 7 we visually check the segmentation result of one test MR image on 2D sagittal slices. In Fig 8 (left part), we compare the segmented surface models of two MR images with the surface models generated from the associated ground truth segmentation. It can be clearly seen that the our method achieved good segmentation results on the test MR images when the results obtained with the present method are compared to the corresponding ground-truth segmentation.

#### 3.3.4 Segmentation results on CT data

For quantitative evaluation of the present method on the 10 CT test images, the Dice, AAD, and HSD between automatic segmentation and ground-truth segmentation are calculated over both 3D volumes and 2D mid-sagittal slices. Table 8 presents the Dice, AAD, and HSD (average of the 5 VBs) of each test image as well as overall mean, std and median values of Dice, AAD, and HSD when calculated on 2D mid-sagittal slices. Similarly, Table 9 presents the Dice, AAD, and HSD of each image as well as overall mean, std and median values of Dice, AAD, and HSD when calculated on 3D volumes. In summary, we achieved a mean Dice of 90.8±8.7%, a mean AAD of 1.0±0.7 mm, and a mean HSD of 4.3±2.2 mm when evaluated on 2D mid-sagittal slices. For 3D evaluation, we achieved a mean Dice of 91.0±7.0%, a mean AAD of 0.9±0.3 mm and a mean HSD of 7.3±2.2 mm.

**Table 8:** Segmentation results when the present method was evaluated on 10 3D CT images with a leave-one-out experiment. The results are calculated on 2D mid-sagittal slices.

Dice overlap coefficient on 2D mid-sagittal slice (%)											
#1	#2	#3	#4	#5	#6	#7	#8	#9	#10	Mean ± STD	Median
92.8	88.2	93.6	94.6	88.4	94.1	87.2	95.9	94.6	78.1	90.8 ± 8.7	93.9
Average absolute distance on 2D mid-sagittal slice (mm)											
#1	#2	#3	#4	#5	#6	#7	#8	#9	#10	Mean ± STD	Median
1.0	0.8	0.8	0.7	1.2	0.7	1.9	0.6	0.9	1.5	1.0 ± 0.7	0.8
Hausdorff distance on 2D mid-sagittal slice (mm)											
#1	#2	#3	#4	#5	#6	#7	#8	#9	#10	Mean ± STD	Median
3.4	4.2	3.2	3.6	7.5	4.0	3.5	2.2	2.0	9.0	4.3 ± 2.2	3.6

**Table 9:** Segmentation results when the present method was evaluated on 10 3D CT images with a leave-one-out experiment. The results are calculated on 3D volumes.

Dice overlap coefficient on 3D volume (%)											
#1	#2	#3	#4	#5	#6	#7	#8	#9	#10	Mean ± STD	Median
91.8	90.7	93.4	93.8	87.0	92.6	90.1	94.5	93.8	82.0	91.0 ± 7.0	93.0
Average absolute distance on 3D volume (mm)											
#1	#2	#3	#4	#5	#6	#7	#8	#9	#10	Mean ± STD	Median
1.0	0.8	0.7	0.8	1.1	0.7	1.3	0.7	0.9	1.0	0.9 ± 0.3	0.8
Hausdorff distance on 3D volume (mm)											
#1	#2	#3	#4	#5	#6	#7	#8	#9	#10	Mean ± STD	Median
5.0	7.3	7.7	6.0	10.3	5.5	10.0	5.8	5.3	10.2	7.3 ± 2.2	6.6

**Fig 9:**
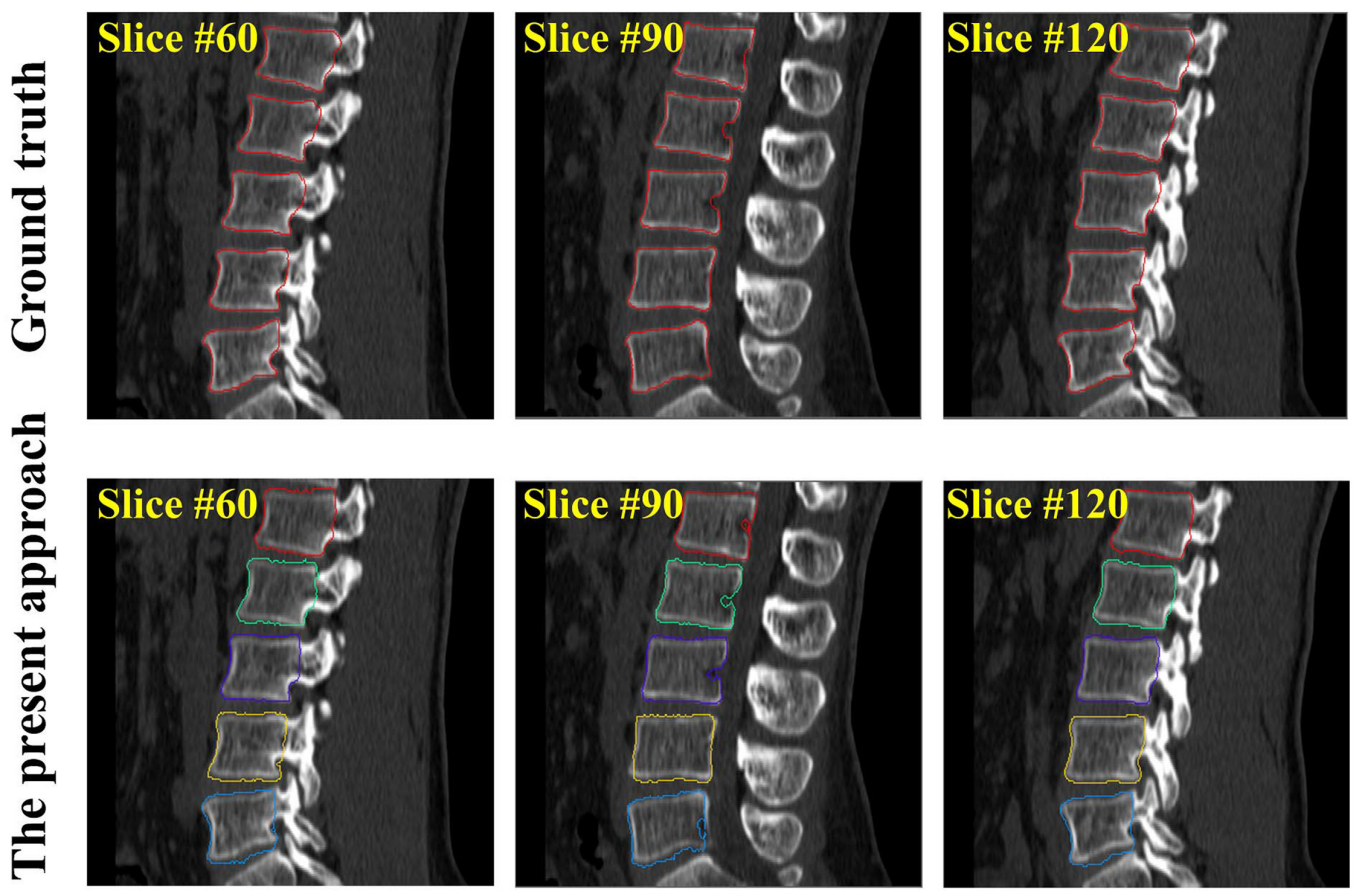
Segmentation results on one test CT image visualized in 2D sagittal slices. The automatic segmentation (the bottom row) are compared with the ground-truth segmentation (the top row).

In Fig 9 we visually check the segmentation results of one test CT image on 2D sagittal slices. In Fig 8 (right part), we compare the segmented surface models of two CT images with the surface models generated from the associated ground truth segmentation. It is worth to note that the second CT data (bottom right image of Fig 8) contains osteophytes in some of the VB regions. Nevertheless, our method successfully identified and segmented all the 5 VB regions in this CT data with a Dice of 90.7%.

#### 3.3.5 Computation Time

When our Matlab implementation was executed on a computer with 3.0 GHz CPU and 12G RAM, the run-time for the present method could be summarized as follows: 1) For experiments conducted on MR images, on average the run-time of the present approach to localize and segment one image was about 2.0 minutes, in which 0.7 minutes for localization and 1.3 minutes for segmentation. 2) For experiments conducted on CT images, on average the run-time of the present approach to localize and segment one image was about 2.3 minutes, in which 0.5 minutes for localization and 1.8 minutes for segmentation.

The computation time for training RF regressors was respectively about 7.4 minutes for a leave-one-out study conducted on the MR images and 9.7 minutes for a leave-one-out study conducted on the CT images. Although the training phase took relatively longer time when compared to the test phase as described above, we only need to perform the training once in our learning-based method. The trained RF regressors can then be used for any future test image.

## 4 Discussions

We presented a fully automatic method to localize and segment VBs from CT/MR images. For localization, a RF regression algorithm is used where we aggregate the votes from a set of randomly sampled image patches to get a probability map of the center of a target VB in a given image. The resultant probability map is further regularized by HMM to eliminate potential ambiguity caused by the neighboring VBs. After the VB center are localized, we segment the VBs by classifying image voxels in an ROI around the VB center. We use a RF classification to estimate the likelihood of a voxel being foreground or background. The estimated likelihood is combined with the prior probability, which is learned from a set of training data, to get the posterior probability of the voxel. The segmentation of the target VB is then done by a binary thresholding of the estimated probability. The present method is validated on both MR and CT images using leave-one-out experiments. Experimental results show that our method achieves accurate results on both MR and CT images.

Compared with the user-supplied methods [3, 6, 7], the present method can achieve VB localization fully automatically without any user-intervention. The automatic strategy has the advantages of reducing measurement time and improving clinical study quality. Our experimental results demonstrated the efficiency and accuracy of the RF regression based method with: 1) average localization time about 0.7 minute for detecting 7 VB regions from a 3D MR image and 0.5 minute for detecting 5 VB regions from a 3D CT image, and 2) a mean localization error of 1.6 mm when evaluated on MR images and 1.9 mm when evaluated on CT images.

To the best of our knowledge, this is the first time to apply RF classification for VB segmentation in CT/MR images. Although there exist works using RF classification for medical image segmentation, they are only specified to segment soft tissues like kidney in CT images [30]. Furthermore, our experimental results demonstrated the accuracy and robustness of the RF classification based method for VB segmentation in CT/MR images. More specifically, the present method achieved a mean Dice of 88.7% when evaluated on 3D MR images and a mean dice of 92.0% when evaluated on 2D mid-sagittal MR slices. In comparison with GT based methods for MR image segmentation, the present method achieved better results. For example, the 2D square-cut method [6] achieved an average Dice of 90.97% while the 3D cube-cut method [20] achieved an average Dice of 81.33%. Nevertheless, due to the fact that different datasets are used in evaluation of different methods, direct comparison of different methods is difficult and should be interpreted cautiously.

Most of the work [4, 5, 16, 21, 40] on spine CT image processing focuses on segmentation and there are a few studies addressing automatic localization of vertebrae in CT scan [8, 17, 40]. Random forest regression was used in both [8] and this study for an automatic localization of vertebrae from CT scans but with different visual feature design. In comparison with the results reported in [8] where an average localization error of 6.06 mm was reported for lumbar vertebrae, our method achieved better results, with an average localization error of 1.9 mm. Again, due to different datasets used in evaluation of different methods, such a comparison should be interpreted cautiously. It is worth to note that the datasets used in [8] are much more diverse than the CT datasets used in our study, which may pose a challenge to their method and partially explain why we have achieved better results. In comparison with other spine CT segmentation methods, the CT segmentation accuracy of the present method is slightly worse than those model-based approaches [4, 5, 21], though the present method achieves a segmentation accuracy on 3D MR images that is comparable to the state-of-the-art spine MRI segmentation methods [3, 6, 15, 20]. For example, evaluated on the same datasets, the method introduced in [21] achieved an average Dice coefficient of 93.6% while the proposed approach achieved an average Dice coefficient of 91.0%. Nevertheless, the present method has the advantage that it can be used to segment VBs from both MR and CT images while most of the state-of-the-art CT segmentation methods are designed for segmenting CT data only.

## 5 Conclusions

In summary, this work has the research highlights as described below:

1. Proposed a fully automatic, unified RF regression and classification framework;
2. Solved the two important problems of localization and segmentation of VB regions from a 3D CT image or a MR image with the unified framework;
3. Validated and evaluated the proposed framework on 10 3D CT data and 23 3D MRI data;
4. Achieved comparable or equivalent segmentation performance to the state-of-the-art methods.
